# Medication Changes Among Older Drivers Involved in Motor Vehicle Crashes

**DOI:** 10.1001/jamanetworkopen.2024.38338

**Published:** 2024-10-09

**Authors:** Andrew R. Zullo, Melissa R. Riester, Adam M. D’Amico, Monika Reddy Bhuma, Marzan A. Khan, Allison E. Curry, Melissa R. Pfeiffer, Seth A. Margolis, Brian R. Ott, Thomas Bayer, Nina R. Joyce

**Affiliations:** 1 Department of Epidemiology, Brown University School of Public Health, Providence, Rhode Island; 2 Department of Health Services, Policy, and Practice, Brown University School of Public Health, Providence, Rhode Island; 3 Center of Innovation in Long-Term Services and Supports, Providence Veterans Affairs Medical Center, Providence, Rhode Island; 4 Center for Gerontology and Health Care Research, Brown University School of Public Health, Providence, Rhode Island; 5 Center for Injury Research and Prevention, Children’s Hospital of Philadelphia, Philadelphia, Pennsylvania; 6 Division of Emergency Medicine, Department of Pediatrics, Perelman School of Medicine, University of Pennsylvania, Philadelphia; 7 Rhode Island Hospital, Providence; 8 Department of Psychiatry and Human Behavior, Alpert Medical School, Brown University, Providence, Rhode Island; 9 Department of Neurology, Alpert Medical School, Brown University, Providence, Rhode Island; 10 Division of Geriatrics and Palliative Medicine, Alpert Medical School, Brown University, Providence, Rhode Island

## Abstract

**Importance:**

Although older adults may use potentially driver-impairing (PDI) medications that can produce psychomotor impairment, little is known about changes to PDI medication use among older adults from the time before to the time after a motor vehicle crash (MVC).

**Objective:**

To quantify use of and changes in PDI medications among older adults before and after an MVC.

**Design, Setting, and Participants:**

This cohort study used linked Medicare claims and police-reported MVC data on 154 096 person-crashes among 121 846 older drivers. Eligible persons were drivers aged 66 years or older, involved in a police-reported MVC in New Jersey from May 1, 2007, through December 31, 2017, and with continuous enrollment in Medicare fee-for-service Parts A and B for at least 12 months and Part D for at least 120 days prior to the MVC. Data were analyzed from January 2022 to May 2024.

**Main Outcomes and Measures:**

Use of benzodiazepines, nonbenzodiazepine hypnotics, opioid analgesics, and other PDI medications in the 120 days before and 120 days after the MVC. Because each person could contribute multiple MVCs during the study period if they met eligibility criteria, the unit of analysis was the number of person-crashes. The proportion of person-crashes after which PDI medications were started, discontinued, or continued was quantified as well.

**Results:**

Among 154 096 eligible person-crashes, the mean (SD) age of the drivers was 75.2 (6.7) years at the time of the MVC. Of 121 846 unique persons, 51.6% were women. In 80.0% of the person-crashes, drivers used 1 or more PDI medications before the crash, and in 81.0% of the person-crashes, drivers used 1 or more PDI medications after the crash. Use of benzodiazepines (8.1% before the crash and 8.8% after the crash), nonbenzodiazepine hypnotics (5.9% before the crash and 6.0% after the crash), and opioid analgesics (15.4% before the crash and 17.5% after the crash) was slightly higher after the MVC. After the MVC, drivers in 2.1% of person-crashes started benzodiazepines and 1.4% stopped benzodiazepines, drivers in 1.2% of person-crashes started nonbenzodiazepine hypnotics and 1.2% stopped nonbenzodiazepine hypnotics, and drivers in 8.4% of person-crashes started opioid analgesics and 6.3% stopped opioid analgesics.

**Conclusions and Relevance:**

This cohort study suggests that most older drivers involved in MVCs did not use fewer PDI medications after crashes than before crashes. Qualitative research of perceived risks vs benefits of PDI medications is necessary to understand the reasons why MVCs do not appear to motivate clinicians to deprescribe PDI medications as a strategy to avert potential harms, including additional MVCs.

## Key Points

**Question:**

How does the use of benzodiazepines, nonbenzodiazepine hypnotics, and opioid analgesics change among older drivers after a motor vehicle crash?

**Findings:**

This cohort study included 154 096 person-crashes among 121 846 older drivers involved in motor vehicle crashes. After the crash, 2.1% of individuals started and 1.4% stopped benzodiazepines, 1.2% started and 1.2% stopped nonbenzodiazepine hypnotics, and 8.4% started and 6.3% stopped opioid analgesics after the crash.

**Meaning:**

This study suggests that most older drivers involved in motor vehicle crashes do not start using fewer potentially driver-impairing medications after crashes than before.

## Teaser

This cohort study quantifies use of and changes in potentially driver-impairing medications (benzodiazepines, nonbenzodiazepine hypnotics, and opioid analgesics) among older adults before and after a motor vehicle crash.

## Introduction

Motor vehicle crashes (MVCs) are a major source of morbidity and mortality for older adults, resulting in nearly 7000 deaths and more than 191 000 nonfatal injuries treated in US emergency departments annually.^1^ Older adults are at increased risk of MVCs in large part due to age-related declines in sensory, cognitive, and physical function, as well as medical conditions that may affect driving (eg, insomnia, Parkinson disease, and arthritis).^2^ In the US alone, there are now more than 60 million adults aged 65 years or older, constituting approximately 17% of the total population, and more than 56 million of them are licensed to drive.^3^ Both the number of older adults and their share of the population are expected to continue to increase, with 65 million drivers among more than 73 million older adults projected by 2030.^4^ The size and the growth of the older adult population have created a need to identify factors that can be used to help lower the risk of MVCs. Because approximately 20% of crash-involved older drivers will have another MVC,^5^ identifying modifiable factors to prevent additional crashes is crucial.

Prescription drug use is a modifiable potential risk factor for MVCs among older adults. Approximately 90% of adults aged 65 years or older take at least 1 prescription medication, and 40% take 5 or more medications.^6,7^ Many of the medications older adults use are potentially driver impairing (PDI) and produce psychomotor impairment.^8,9,10,11,12,13^ For example, driving simulator and related tests (eg, closed-course driving assessments)^14,15,16^ have shown that sedative-hypnotic drugs produce a dose-dependent impairment in driving-related psychomotor skills, including reaction time,^17^ stimulus detection,^18^ and hand-eye coordination.^17^ A single dose may impair road-tracking ability during uninterrupted high-speed highway driving.^19^ Similar adverse effects have been documented in controlled laboratory and other test settings for barbiturates, antidepressants, sedating antihistamines, and opioids.^17,20,21^ In addition, observational studies have identified increased risks of MVCs among users of several medication classes.^22,23^

Little is known about older adults’ medication use patterns before and after an MVC, including whether MVCs might spur clinicians to change PDI medication use. Previous work found that most crash-involved older drivers were exposed to multiple PDI medications before an MVC and that there was no net change in the number of PDI medication classes used after MVCs.^24^ However, that study focused on the prevalence of polypharmacy with PDI medications in the precrash and postcrash periods overall and did not examine how the daily probability of PDI medication use changed across the time period closely surrounding an MVC, which could provide further insights into the associations between PDI medication prescribing and crash events. An MVC could act as a trigger for clinician intervention, particularly through deprescribing, which could be initiated soon after the MVC to lower the risk of future MVCs. Elucidating how PDI medication use changes in association with an MVC is necessary to inform our understanding of how to effectively balance autonomy and safety among older adults (ie, ensuring safe mobility while maintaining independence).

This study aimed to (1) quantify the use of benzodiazepines, nonbenzodiazepine hypnotics, and opioid analgesics in the time period closely surrounding an MVC and (2) assess changes in the use of these medication classes from before to after an MVC, including starting and stopping medications. We chose to focus on these 3 medication classes because they have the greatest evidence sufficiency to suggest that they confer a higher risk of MVC and have been highlighted by the National Highway Traffic Safety Administration.^22,25,26^

## Methods

### Study Design and Data Sources

In this cohort study, we linked fee-for-service Medicare claims with the New Jersey Safety and Health Outcomes (NJ-SHO) warehouse for crash-involved older drivers. Data from NJ-SHO and Medicare were linked at the level of the driver using a strict matching algorithm that included last name, birth date, sex, and zip code. Medicare data included the Medicare Beneficiary Summary File (MBSF), Medicare Provider Analysis and Review (MedPAR) inpatient claims, Medicare Carrier professional service claims, and Medicare Part D pharmacy dispensing claims. The NJ-SHO warehouse used data from various statewide administrative sources, such as police-reported crashes and driver licensing information.^27^ The study was approved by the Brown University institutional review board, which waived informed consent because the data were deidentified. This study followed the Strengthening the Reporting of Observational Studies in Epidemiology (STROBE) reporting guideline for cohort studies. Additional information about the data and methods is available in the Brown Digital Repository.^28^

### Study Population

Eligible persons were aged 66 years or older and were involved in a police-reported MVC in New Jersey as a driver from May 1, 2007, through December 31, 2017. A crash is reportable in New Jersey if it results in an injury or death of any person or more than $500 in property damage to any 1 person.^27^ We included individuals who were licensed in New Jersey at any point between 2004 and 2018 and who were not driving with a permit on the day of the MVC, who were continuously enrolled in Medicare fee-for-service Parts A and B for at least 12 months prior to the crash and continuously enrolled in Part D for 120 days prior to the crash, and who were not enrolled in Medicare Advantage in the 12 months prior to the crash. Medicare Advantage beneficiaries were excluded because missing and inaccurate encounter data were a concern.^29^ A person could be involved in more than 1 MVC during the study period, so analyses were conducted at the event or person-crash level to maximize the generalizability of our study findings.

### PDI Medication Use

Patterns of PDI medication use in the 120 days prior to the MVC and the 120 days after the MVC were examined. We selected a 120-day period to ensure that medication dispensings with a 3-month supply prior to the MVC were captured. Analyses were focused primarily on the use of benzodiazepines, nonbenzodiazepine hypnotics, and opioid analgesics.^2,30^ We also examined any use of PDI medication classes (eTable 1 in Supplement 1) with evidence to support a potential for increased MVC risk, including those identified in the American Geriatrics Society Clinician’s Guide to Assessing and Counseling Older Drivers.^2,22,30,31,32,33,34,35,36,37,38,39^

We constructed medication use episodes to estimate when drivers in person-crashes had PDI medications available in the pre-MVC and post-MVC periods. The start of a medication use episode was the date of medication dispensing. Drivers in person-crashes were considered to have the drug available until the end of the medication use episode, which was the episode start date plus the days of medication supplied plus a grace period of 50% of the number of days of supply (ie, the end of a medication use episode for a dispensing with 30 days of supply would be 45 days after the date of medication dispensing).

Drivers in person-crashes were considered to have used medication in the pre-MVC period if they had medication available for at least 1 day between day 1 and day 120 before the MVC, while having medication available for at least 1 day between day 0 and day 120 after the crash defined use during the post-MVC period. Individuals were considered to have started a medication after a crash if there was no use of the medication class in the pre-MVC period but use in the post-MVC period. Individuals were considered to have stopped a medication after a crash if there was use of the medication class in the pre-MVC period but no use in the post-MVC period. Individuals were considered to have continued a medication after a crash if there was use of the medication class in the pre-MVC and post-MVC periods.

### Participant Characteristics

Demographic characteristics (age, sex, and race and ethnicity [Research Triangle Institute race code; American Indian or Alaska Native, Asian or Other Pacific Islander, Hispanic, non-Hispanic Black, non-Hispanic White, and unknown or other race or ethnicity (other includes any other race or ethnicity not included in the other enumerated categories)]) and dual Medicare and Medicaid enrollment information were obtained from the MBSF. Race and ethnicity (as measured by the race code as a single variable) were assessed to describe the study population so that readers could evaluate the generalizability of the study findings to target populations of interest. MedPAR, Part B Carrier, and skilled nursing facility claims in the 12 months prior to the crash were used to quantify the Gagne Combined Comorbidity Index as a measure of multimorbidity (range, −2 to 26, where higher scores indicate greater multimorbidity).^40^ We also ascertained the total number of unique drug dispensings in the pre-MVC period and health care utilization in the 7 days after MVC (hospital admission, intensive care unit admission, and emergency department visit). At-fault status for the MVC was defined by the driver committing at least 1 driver-initiated action (eg, unsafe speed or driver inattention) that contributed to the crash, as determined by the investigating police officer.^41,42,43^

### Statistical Analysis

Analyses were conducted from January 2022 to May 2024. We calculated the proportion of person-crashes with drivers with any prescription medication use, PDI medication use, or use of benzodiazepines, nonbenzodiazepine hypnotics, or opioid analgesics in the pre-MVC and post-MVC periods. The proportion of person-crashes with drivers who started, discontinued, or continued PDI medications after the crash was quantified. We also visualized the daily probability of having PDI medications available on any given day in the 120 days before to the 120 days after the crash date by dividing the number of users of PDI medications by the total number of person-crashes in the study population. The primary analyses were conducted separately for benzodiazepines, nonbenzodiazepine hypnotics, and opioid analgesics. Secondary analyses were also performed for a composite of all PDI medication classes. Data were analyzed using SAS, version 9.4 (SAS Institute Inc).

## Results

### Study Population

The study population included older drivers with 154 096 eligible person-crashes (eFigure 1 in Supplement 1). The mean (SD) age of the drivers was 75.2 (6.7) years at the time of the MVC. Of 121 846 unique individuals, 51.6% were women and 48.4% were men; 1.0% were American Indian or Alaska Native, 3.2% were Asian or Other Pacific Islander, 4.8% were Hispanic, 6.9% were non-Hispanic Black, 83.2% were non-Hispanic White, and 0.8% were of unknown or other races or ethnicities (Table 1). Overall, the mean (SD) Gagne Combined Comorbidity Index prior to the crash was 1.4 (2.3). Drivers in person-crashes had a mean (SD) of 5.4 (4.0) unique drug dispensings in the precrash period, 1.9% were hospitalized within 7 days after the crash, 7.0% had an emergency department visit within 7 days after the crash, and 57.8% were cited as having at least 1 at-fault driving behavior for the crash.

**Table 1.** Characteristics of Older Adults Who Were Drivers Involved in a Motor Vehicle Crash

Characteristic	No. (%)
**Person-crashes^a^**	
Total No.	154 096
Age at crash, mean (SD), y	75.2 (6.7)
Age category, y	
66-69	37 867 (24.6)
70-74	42 456 (27.6)
75-79	32 462 (21.1)
80-84	24 078 (15.6)
≥85	17 233 (11.2)
Medicaid dual eligibility at any point in year before crash	10 992 (7.1)
Gagne Combined Comorbidity Index, mean (SD)^b^	1.4 (2.3)
Total unique drug dispensings from 120 d before the crash to 1 d before the crash, mean (SD)	5.4 (4.0)
Hospitalized within 7 d after the crash	2959 (1.9)
ICU admissions within 7 d after the crash	1365 (0.9)
Emergency department visit within 7 d after the crash	10 788 (7.0)
At-fault for motor vehicle crash^c^	89 066 (57.8)
**Unique individuals** ^d^	
Total No.	121 846
Sex	
Female	62 915 (51.6)
Male	58 931 (48.4)
Race and ethnicity	
American Indian or Alaska Native	1213 (1.0)
Asian or Other Pacific Islander	3887 (3.2)
Hispanic	5888 (4.8)
Non-Hispanic Black	8430 (6.9)
Non-Hispanic White	101 422 (83.2)
Unknown or other^e^	976 (0.8)

Abbreviation: ICU, intensive care unit.

^a^ A person could be involved in more than 1 motor vehicle crash during the study period, so information is presented at the person-crash level as measures could vary between crashes for a particular person.

^b^ Range, −2 to 26, where higher scores indicate greater multimorbidity.

^c^ A driver was classified as being responsible for the crash if they committed at least 1 driver-initiated action (eg, unsafe speed or driver inattention) that contributed to the crash as determined by the investigating police officer.

^d^ Time-invariant characteristics are reported at the person level.

^e^ “Other” is an original category of the Research Triangle Institute race code in the Medicare Beneficiary Summary File and includes any other race or ethnicity not included in the other enumerated categories.

### Initiation and Discontinuation of PDI Medications

In 80.0% of the person-crashes, drivers used 1 or more PDI medications before the crash, and in 81.0% of the person-crashes, drivers used 1 or more PDI medications after the crash (Table 2). The most prevalent PDI medications classes used prior to the crash were antihypertensives, antidepressants, and opioids (eTable 1 in Supplement 1). Drivers in most person-crashes (77.6%) used 1 or more PDI medications both before and after the MVC (eFigure 2 in Supplement 1).

**Table 2.** Medication Use Among Older Drivers in the 120 Days Before and 120 Days After a Motor Vehicle Crash

Medication use	Person-crashes, No. (%) (N = 154 096)	
	Before crash	After crash
Any medication	142 072 (92.2)	142 321 (92.4)
Any potentially driver impairing medication^a^	123 304 (80.0)	124 796 (81.0)
Benzodiazepine	12 503 (8.1)	13 533 (8.8)
Nonbenzodiazepine hypnotic	9114 (5.9)	9227 (6.0)
Opioid analgesic	23 727 (15.4)	26 917 (17.5)

^a^ Prevalence of individual potentially driver impairing medication classes are described in eTable 1 in Supplement 1.

**Figure 1.**
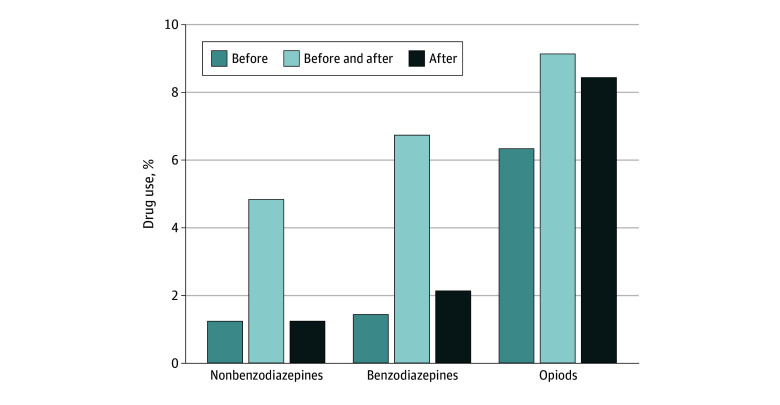
Initiation and Discontinuation of Benzodiazepines, Nonbenzodiazepine Hypnotics, and Opioid Analgesics After Motor Vehicle Crash Comparison of medication use in the precrash period (120 days before the crash date) with the postcrash period (120 days after) among older drivers involved in motor vehicle crashes (N = 154 096 person-crashes). “Before” indicates users who discontinued the drug prior to or after the crash, “before and after” indicates users who continued the drug after the crash, and “after” indicates nonusers before the crash who started the drug only after the crash.

Use of benzodiazepines (8.1% before the crash and 8.8% after the crash), nonbenzodiazepine hypnotics (5.9% before the crash and 6.0% after the crash), and opioid analgesics (15.4% before the crash and 17.5% after the crash) was slightly higher after the MVC (Table 2). Overall, 2.1% of drivers in person-crashes started benzodiazepines after the MVC, 1.2% started nonbenzodiazepine hypnotics, and 8.4% started opioid analgesics, while 1.4% stopped benzodiazepines, 1.2% stopped nonbenzodiazepine hypnotics, and 6.3% stopped opioid analgesics after the MVC (Figure 1). Among users of benzodiazepines (n = 12 503), nonbenzodiazepine hypnotics (n = 9114), and opioids (n = 23 727) in the precrash period, 17.2% of benzodiazepine users, 19.6% of nonbenzodiazepine hypnotic users, and 41.2% of opioid users discontinued use after the crash (eTable 2 in Supplement 1). Among users of benzodiazepines (n = 13 533), nonbenzodiazepine hypnotics (n = 9227), and opioids (n = 26 917) in the postcrash period, 23.5% of benzodiazepine users, 20.5% of nonbenzodiazepine hypnotic users, and 48.1% of opioid users initiated use after the crash.

### Probability of PDI Medication Use Before and After Crash

**Figure 2.**
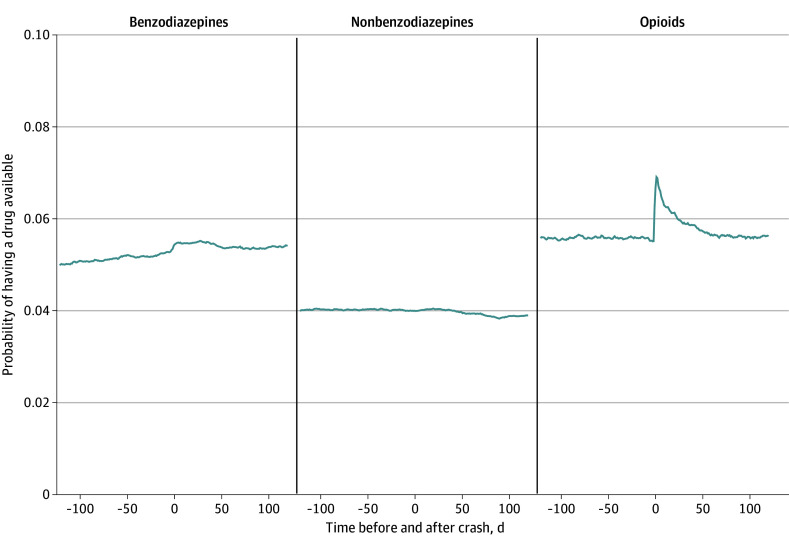
Probability of Using Benzodiazepines, Nonbenzodiazepine Hypnotics, and Opioid Analgesics Before and After Motor Vehicle Crash Probability of having medication available on any given day in the 120 days before to the 120 days after the crash date among older drivers involved in motor vehicle crashes (N = 154 096 person-crashes).

The daily probability of having any PDI medication available varied minimally throughout the precrash and postcrash periods (eFigure 3 in Supplement 1). Use of benzodiazepines increased slightly around the time of the crash and decreased across the postcrash period, although the probability of having benzodiazepines available was greater in the postcrash period than the precrash period (Figure 2). There was little change in the probability of having nonbenzodiazepine hypnotics available in the precrash and postcrash periods. A spike in the probability of having opioids available occurred on the day of the crash and gradually decreased over time, nearing precrash probability levels around day 50 after the crash.

## Discussion

In this cohort study of older drivers involved in MVCs, we found that most clinicians did not stop PDI medications for older drivers who experienced an MVC. Nearly 80% of person-crashes involved the use of 1 or more PDI medications both in the 120 days prior to and in the 120 days after the MVC. Use of benzodiazepines and nonbenzodiazepine hypnotics changed slightly after the MVC, while changes in the use of opioids were more pronounced. Overall, drivers in 2.1% of person-crashes started benzodiazepines and 1.4% stopped benzodiazepines, 1.2% started nonbenzodiazepine hypnotics and 1.2% stopped nonbenzodiazepine hypnotics, and 8.4% started opioid analgesics and 6.3% stopped opioid analgesics after the MVC. Research is needed to understand if clinicians are missing an opportunity to reduce future MVC risk through deprescribing.

Numerous studies have reported that psychoactive medications such as benzodiazepines, nonbenzodiazepine hypnotics, and opioids can impair driving ability and may increase the risk for MVCs.^8,9,10,11,12,13,14,15,16,17,18,19,20,21,22,31,32,33,34,35,36,37,38,39,44,45^ However, studies examining the association of reducing drivers’ exposure to these medications with subsequent MVC outcomes are scarce. Consequently, clinician guidance on how to manage pharmacotherapy for older adults who take PDI medications and experience driving impairment or MVCs is limited. This study extends prior work by showing few changes in PDI use after an MVC, highlighting the need for safety interventions to modify PDI prescribing after an MVC.

In addition to advising patients about appropriate use and warnings while driving at the time of initial prescription, 1 potential strategy to further reduce the risk of subsequent MVCs is for clinicians to perform a medication review and decrease older drivers’ exposure to PDI medications through dose reduction, deprescribing, or medication switching when appropriate. We found that deprescribing PDI medications is not a common practice after an MVC. Similarly, a prior study reported that approximately 65% of crash-involved older drivers had no net change in the number of PDI medications after MVC.^24^ A potential barrier to taking action is that many clinicians may not be aware that their patients have been involved in an MVC. There are no well-developed mechanisms that are widely used to ensure that clinicians are notified when their patients are involved in an MVC. If a person does not seek health care for issues related to the MVC or does not mention the crash at a subsequent routine clinician visit, it is unlikely that their clinician would know that the crash occurred. In our study, only 1.9% of drivers in person-crashes were hospitalized, and 7.0% had an emergency department visit within 7 days after the MVC. Future studies using survey data, qualitative research, and mixed-methods research are necessary to understand how often clinicians consider modifying PDI medications for patients who experienced MVCs and the reasons for not deprescribing these medications. Intervention studies examining the association of deprescribing PDI medications with subsequent MVCs are also needed to understand the effectiveness of post-MVC medication review and deprescribing.

Several alternative explanations exist for the rarity of deprescribing PDI medications after an MVC. Although many clinicians may be unaware that their patients have been involved in an MVC, those who are aware might not know about the associations between certain medications and MVC risk. Even if clinicians are aware, they may have other priorities that take precedence over deprescribing efforts, such as managing other comorbidities or addressing more immediate health concerns. In addition, patients may not be aware of the associations between their medications and increased MVC risk. Even if patients are aware, they might prefer to continue these medications due to perceived benefits or fear of discontinuation if they inform their clinician about the MVC. Although research is necessary to identify the prevalence of each of these possible explanations, future deprescribing interventions might need to focus on improving the awareness of health care professionals and patients about the risks associated with PDI medications and the potential benefits associated with deprescribing, as well as establishing better communication channels to ensure that MVCs are reported and discussed during clinical visits.

A greater proportion of drivers in person-crashes started benzodiazepines and opioids than stopped those medications after the MVC. This is likely because patients started benzodiazepines and opioids to manage pain and other sequelae resulting from the MVC, such as anxiety and muscle strain. However, we could not examine the appropriateness of PDI medication prescriptions in our study because information on medication indication was not available in our data. Thus, further research is necessary to estimate how often it would be clinically appropriate to intervene on PDI medication prescribing.

### Limitations

This study has several potential limitations. First, our results may not generalize well to those without Medicare fee-for-service insurance (ie, Medicare Advantage), individuals who reside outside of the New Jersey area, or crashes that did not result in a police officer responding to and reporting the crash. Second, there is no accepted consensus for defining PDI medications and their relative risk for use among drivers. Varying study designs are used to assess whether medications are PDI (eg, driving simulator or epidemiologic), although there are likely differences in the validity of these different approaches. We believe our list of PDI medications is comprehensive; however, there may be other medication classes that could have been included as having the potential to impair driving ability. Third, the 120-day post-MVC period used in our analysis might not capture instances in which clinicians discontinued PDI medications more than 120 days after the MVC, thereby misclassifying some discontinuations as continuations. Fourth, given the nature of our prescription drug claims data, it was not possible to confirm medication administrations or adherence to PDI medications. This may have resulted in misclassification of medication exposure where (1) drivers in person-crashes were considered to have used PDI medications throughout medication use episodes, although they may have missed doses or discontinued use prior to the estimated end date, and (2) drivers in person-crashes were considered to have not used PDI medications outside of medication use episodes, although they may have taken PDI medications that were left over from a prior dispensing. In addition, we could not assess the timing of medication administrations in association with driving and cannot report on whether older adults moderated their driving when taking PDI medications.

## Conclusions

In this cohort study, we found that 77.6% of older drivers involved in MVCs used 1 or more PDI medication both before and after the crash. Similarly, there were only slight changes in the use of benzodiazepines and nonbenzodiazepine hypnotics in the precrash and postcrash periods. Future research should examine the evidence for PDI medications and clinicians’ awareness of that evidence when considering the benefits vs risks associated with deprescribing PDI medications after a crash.

## Notes

Supplement 1. **eFigure 1.** Study Cohort Flow Diagram **eFigure 2.** Within-Person Patterns of Initiation and Discontinuation of Potentially Driver Impairing Medications Among Older Drivers Involved in Motor Vehicle Crashes, 120 Days Before and 120 Days After the Crash Date, N= 154,096 Person-Crashes **eFigure 3.** Probability of Using Any Potentially Driver Impairing Medication Class Among Older Drivers Involved in Motor Vehicle Crashes, 120 Days Before Through 120 Days After the Crash Date, N= 154,096 Person-Crashes **eTable 1.** Prevalence of Potentially Driver Impairing Medications Prior to Motor Vehicle Crash **eTable 2.** Use of Non-Benzodiazepine Hypnotics, Benzodiazepines, and Opioid Analgesics Among Older Drivers Involved in Motor Vehicle Crashes, Before and After Crash, N= 154,096 Person-Crashes

Supplement 2.
Data Sharing Statement
