# Error in Conflict of Interest Disclosures

**DOI:** 10.1001/jamanetworkopen.2024.51384

**Published:** 2024-11-22

For the Original Investigation “Medication Changes Among Older Drivers Involved in Motor Vehicle Crashes”^1^ that was published on October 9, 2024, in *JAMA Network Open*, the disclosure statement for coauthor Marzan A. Khan has been removed (ie, grants from Sanofi were removed). The article has been corrected.^1^
