# Supplementary material for: Medication Changes Among Older Drivers Involved in Motor Vehicle Crashes
Source: JAMA Netw Open. Author manuscript; Available in PMC 2024 Dec 19. (PMC11581636; doi:10.1001/jamanetworkopen.2024.38338)
Supplement: Supplementary Online Material — eFigure 1. Study Cohort Flow Diagram eFigure 2. Within-Person Patterns of Initiation and Discontinuation of Potentially Driver Impairing Medications Among Older Drivers Involved in Motor Vehicle Crashes, 120 Days Before and 120 Days After the Crash Date, N= 154,096 Person-Crashes eFigure 3. Probability of Using Any Potentially Driver Impairing Medication Class Among Older Drivers Involved in Motor Vehicle Crashes, 120 Days Before Through 120 Days After the Crash Date, N= 154,096 Person-Crashes eTable 1. Prevalence of Potentially Driver Impairing Medications Prior to Motor Vehicle Crash eTable 2. Use of Non-Benzodiazepine Hypnotics, Benzodiazepines, and Opioid Analgesics Among Older Drivers Involved in Motor Vehicle Crashes, Before and After Crash, N= 154,096 Person-Crashes [file NIHMS2033588-supplement-Supplementary_Online_Material.pdf]

Supplementary Online Content

Zullo AR, Riester MR, D'Amico AM, et al. Medication changes among older drivers involved in motor vehicle crashes. *JAMA Netw Open.* 2024;7(10):e2438338. doi:10.1001/jamanetworkopen.2024.38338

eFigure 1. Study Cohort Flow Diagram
eFigure 2. Within-Person Patterns of Initiation and Discontinuation of Potentially Driver Impairing Medications Among Older Drivers Involved in Motor Vehicle Crashes, 120 Days Before and 120 Days After the Crash Date, N= 154,096 Person-Crashes
eFigure 3. Probability of Using Any Potentially Driver Impairing Medication Class Among Older Drivers Involved in Motor Vehicle Crashes, 120 Days Before Through 120 Days After the Crash Date, N= 154,096 Person-Crashes
eTable 1. Prevalence of Potentially Driver Impairing Medications Prior to Motor Vehicle Crash
eTable 2. Use of Non-Benzodiazepine Hypnotics, Benzodiazepines, and Opioid Analgesics Among Older Drivers Involved in Motor Vehicle Crashes, Before and After Crash, N= 154,096 Person-Crashes

This supplementary material has been provided by the authors to give readers additional information about their work.

© 2024 Zullo AR et al. *JAMA Network Open.*

eFigure 1. Study Cohort Flow Diagram

Motor vehicle crashes in New Jersey
01/01/2007 – 12/31/2017
N= 5,989,959 Person-crashes

**Exclusions**	**Person-Crashes**
-Never licensed in New Jersey	1,653,627
-<66 years on crash date	3,919,477
-Driver did not link to Medicare	25,519
-Medicare Advantage insurance in the 12 months prior to crash	66,782
-Not continuously enrolled in Medicare Parts A and B for at least 12 months prior to crash	46,462
-Not continuously enrolled in Part D 120 days prior to crash	121,256
-Crash prior to 5/1/2007	2,740

**Final Population Totals**	**Person-Crashes**
Total	154,096

© 2024 Zullo AR et al. *JAMA Network Open.*

eFigure 2. Within-Person Patterns of Initiation and Discontinuation of Potentially Driver Impairing Medications Among Older Drivers Involved in Motor Vehicle Crashes, 120 Days Before and 120 Days After the Crash Date, N= 154,096 Person-Crashes

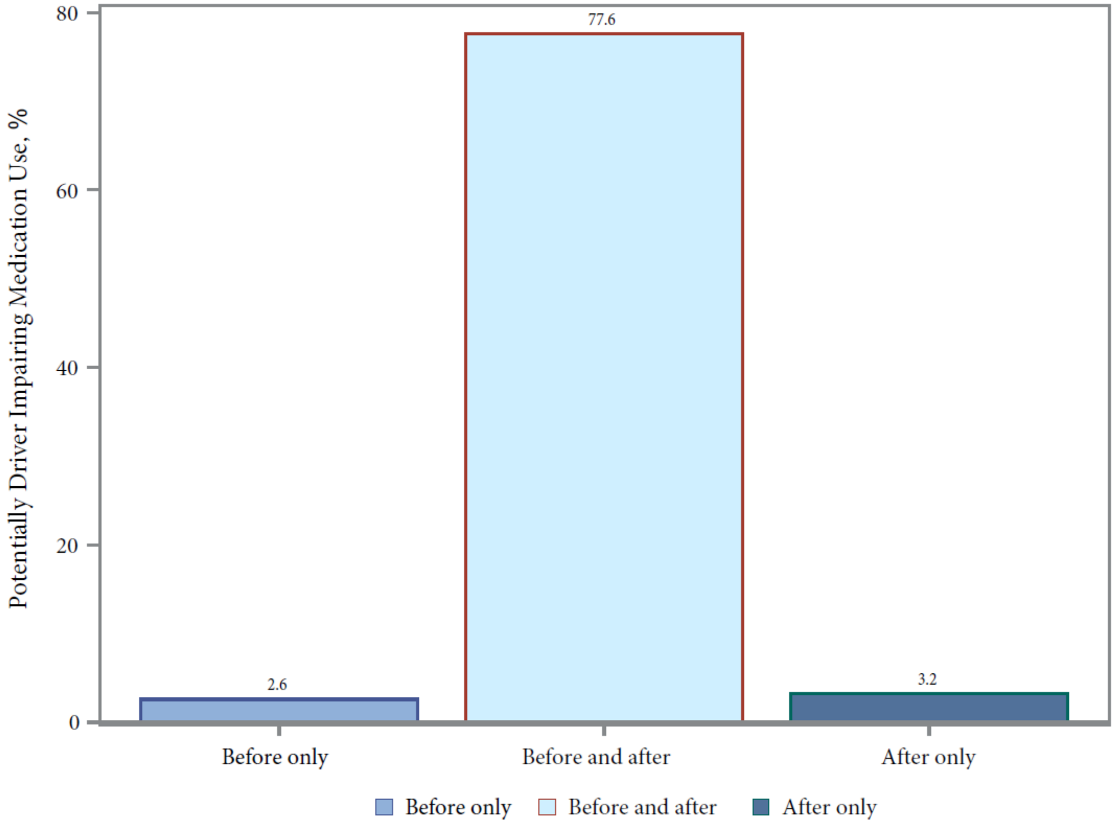

Note: The “Before only” group represents person-crashes who had one or more potentially driver impairing medications “on hand” in the pre-motor vehicle crash (MVC) period and no potentially driver impairing medications “on hand” in the post-MVC period. The “Before and after” group represents person-crashes that had one or more potentially driver impairing medications “on hand” in both the pre-MVC and post-MVC periods. The “After only” group represents person-crashes with no potentially driver impairing medications “on hand” in the pre-MVC period and one or more potentially driver impairing medications “on hand” in the post-MVC period. Percentages are calculated among all person-crashes in the study population, including non-users of any potentially driver impairing medication in the pre-MVC and post-MVC periods, and therefore do not sum to 100%.

© 2024 Zullo AR et al. *JAMA Network Open.*

eFigure 3. Probability of Using Any Potentially Driver Impairing Medication Class Among Older Drivers Involved in Motor Vehicle Crashes, 120 Days Before Through 120 Days After the Crash Date, N= 154,096 Person-Crashes

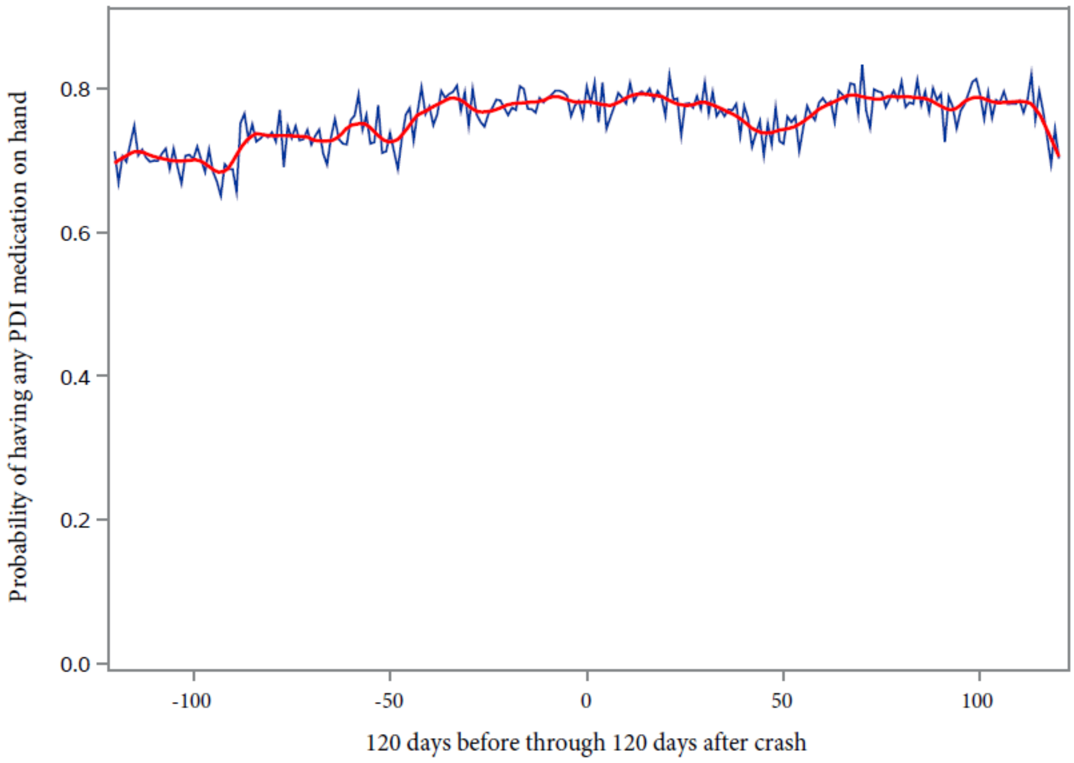

Note: Potentially driver impairing medication classes are outlined in eTable 1.
Abbreviation: PDI, potentially driver impairing.

© 2024 Zullo AR et al. *JAMA Network Open.*

eTable 1. Prevalence of Potentially Driver Impairing Medications Prior to Motor Vehicle Crash

**Drug Class and Putative Mechanism for Increased Crash Risk**	**Prevalence of Use Prior to Crash, No. (%) N= 154,096 person-crashes**
Sedation, impaired cognition, or related mechanisms	
Antidepressants	26,858 (17.4)
Atypical	4,025 (2.6)
Selective serotonin reuptake inhibitors	17,673 (11.5)
Serotonin and norepinephrine reuptake inhibitors	4,986 (3.2)
Tricyclic antidepressants	2,724 (1.8)
Serotonin antagonist and reuptake inhibitors	2,291 (1.5)
Monoamine oxidase inhibitors	29 (0.02)
Antiepileptics	16,105 (10.5)
Antihistamine	7,916 (5.1)
Antiparkinsonian drugs	2,489 (1.6)
Dopamine receptor agonists	1,555 (1.0)
Levodopa	1,111 (0.7)
Muscarinic receptor antagonists for Parkinson’s	147 (0.1)
Monoamine oxidase-B inhibitors for Parkinson’s	258 (0.2)
Catechol-O-methyltransferase inhibitors	59 (0.04)
Antipsychotics	2,264 (1.5)
Atypical / Later-generation	2,060 (1.3)
Typical / Early-generation	240 (0.2)
Anxiolytics	13,238 (8.6)
Barbiturates	1034 (0.7)
Benzodiazepines[a]	12,503 (8.1)
Muscarinic receptor antagonists for overactive bladder	5,952 (3.9)
Non-benzodiazepine hypnotics[a]	9,114 (5.9)
Opioid analgesics[a,b]	23,727 (15.4)
Skeletal muscle relaxants[b]	4,393 (2.8)
Other putative mechanisms	
Antihyperglycemics (via hypoglycemia)	17,743 (11.5)
Insulins	7,038 (4.6)
Sulfonylureas	12,472 (8.1)
Antihypertensives (via hypotension)	106,923 (69.4)
Angiotensin converting enzyme inhibitors	380,99 (24.7)
Angiotensin II receptor blockers	34,623 (22.5)
Alpha blockers for hypertension	4,313 (2.8)
Alpha blockers for benign prostatic hyperplasia	12,986 (8.4)
Beta blockers	54,559 (35.4)
Calcium channel blockers	39,279 (25.5)
Centrally acting agents	2,258 (1.5)
Loop diuretics	15,604 (10.1)
Potassium-sparing diuretics	7,068 (4.6)
Thiazide and thiazide-like diuretics	36,587 (23.7)
Vasodilators	1,628 (1.1)
Stimulants (via aggression)	731 (0.5)

[a]Subset of drugs with the greatest evidence sufficiency to suggest that they confer a higher risk of motor vehicle crash. Evidence sufficiency defined as in Murad MH et al. J Clin Epidemiol. 2021 Jul;135:170-175.
[b]Subset of drugs that are most commonly used to treat the sequelae (i.e., pain) of a crash.
Note: Individual medications included in each medication class are reported in the additional study documentation available in the Brown Digital Repository (https://doi.org/10.26300/nnws-d910).

© 2024 Zullo AR et al. *JAMA Network Open.*

eTable 2. Use of Non-Benzodiazepine Hypnotics, Benzodiazepines, and Opioid Analgesics Among Older Drivers Involved in Motor Vehicle Crashes, Before and After Crash, N= 154,096 Person-Crashes

	Before Crash			After Crash		
**Drug Class**	**Person-crashes with drug available before crash, No.**	**Those who discontinued the drug after crash, No. (%)**	**Those who continued the drug after crash, No. (%)**	**Person-crashes with drug available after crash, No.**	**Those who started the drug after crash, No. (%)**	**Those who continued the drug after crash, No. (%)**
Benzodiazepine	12,503	2,146 (17.2)	10,357 (82.8)	13,533	3,176 (23.5)	10,357 (76.5)
Non-benzodiazepine hypnotic	9,114	1,783 (19.6)	7,331 (80.4)	9,227	1,896 (20.5)	7,331 (79.5)
Opioid analgesic	23,727	9,768 (41.2)	13,959 (58.8)	26,917	12,958 (48.1)	13,959 (51.9)

© 2024 Zullo AR et al. *JAMA Network Open.*
