# Supplementary material for: Medication Changes Among Older Drivers Involved in Motor Vehicle Crashes
Source: JAMA Netw Open. Author manuscript; Available in PMC 2024 Dec 19. (PMC11581636; doi:10.1001/jamanetworkopen.2024.38338)
Supplement: Supplement 2 Data Sharing Statement — Data Sharing Statement [file NIHMS2033588-supplement-Supplement_2_Data_Sharing_Statement.pdf]

Data Sharing Statement

Zullo. Medication Changes Among Older Drivers Involved in Motor Vehicle Crashes. *JAMA Netw Open*. Published October 09, 2024. doi:10.1001/jamanetworkopen.2024.38338

Data
Data available: Yes
Data types: Data dictionary, Other (please specify)
Additional Information: Software code
How to access data: https://doi.org/10.26300/nnws-d910
When available: With publication

Supporting Documents
Document types: None

Additional Information
Who can access the data: The use of data from the Center for Medicare and Medicaid Services (CMS) was covered under the strict terms of a Data Use Agreement (DUA) and individual-level data cannot be shared. Researchers interested in accessing CMS data for their own work should visit the Research Data Assistance Center (ResDAC) at www.resdac.org to get started. A data dictionary and software code are available to any researcher through the Brown Digital Repository (https://doi.org/10.26300/nnws-d910).
Types of analyses: Not applicable.
Mechanisms of data availability: Not applicable. Researchers interested in accessing CMS data for their own work should visit the Research Data Assistance Center (ResDAC) at www.resdac.org to get started.
